# Supplementary material for: Heparan Sulfate Controls Nanoscale Assembly of GPC3-Wnt Receptor Complexes
Source: bioRxiv. 2026 Jul 22:2026.07.21.739947. Preprint. [Version 1] doi: 10.64898/2026.07.21.739947 (PMC13419504; doi:10.64898/2026.07.21.739947)
Supplement: 5 [file NIHPP2026.07.21.739947v1-supplement-5.pdf]

bioRxiv preprint doi: https://doi.org/10.64898/2026.07.21.739947; this version posted July 22, 2026. The copyright holder for this preprint (which was not certified by peer review) is the author/funder. This article is a US Government work. It is not subject to copyright under 17 USC 105 and is also made available for use under a CC0 license.

Figure S1. Confocal microscopy for Wnt3a binding visualization on HCC cell surface. Hep3B-SC22-FZD1-mCherry cells were transfected with Cerulean-GPC3-WT or ΔHS for 36 hours and then incubated with EGFP-Wnt3a conditioned supernatant for 40 min. The cells were visualized with Leica Stellaris 8 FLIM microscope, 63x.

Figure S2. Schematic of FRAP renormalization and curve fitting. A. To extract quantitative values from FRAP curves, we must first measure the recovery curve for a completely immobile protein, such as H2B in fixed cells. The depth of the bleach is considered the Baseline with which other FRAP curves are compared against. B. Experimental FRAP curves will then lie between Baseline and 1, which represents full recovery. C. All curves are then renormalized, such that the Baseline becomes zero. D. Fitting the FRAP curve to an exponential recovery result in the parameters describing the recovery. These parameters consist of: the fast-mobile fraction, F_0, which is estimated from the height of the first point in the curve; the slow-mobile fraction, F_S, which is estimated from the difference between the plateau in the curve and the initial point; the immobile fraction, F_{imm}, which is equal to $1 – (F_0 + F_S)$; and the half-time of recovery, $T_{1/2}$.

Figure S3. Comparison of YP7-labeled GPC3 on the cell membrane. A. Identification of high dense molecular raft by auto-thresholding of channel 488. The corresponding YP7-ATTO647N labeled GPC3 single molecules were present to the right. Scale bars: 2 µm. B. Bound fraction of GPC3 molecules in the high dense molecular raft and low dense area. The GPC3 molecules were analyzed with residence analysis and were compared in high dense molecular and low dense molecular area.

Figure S1

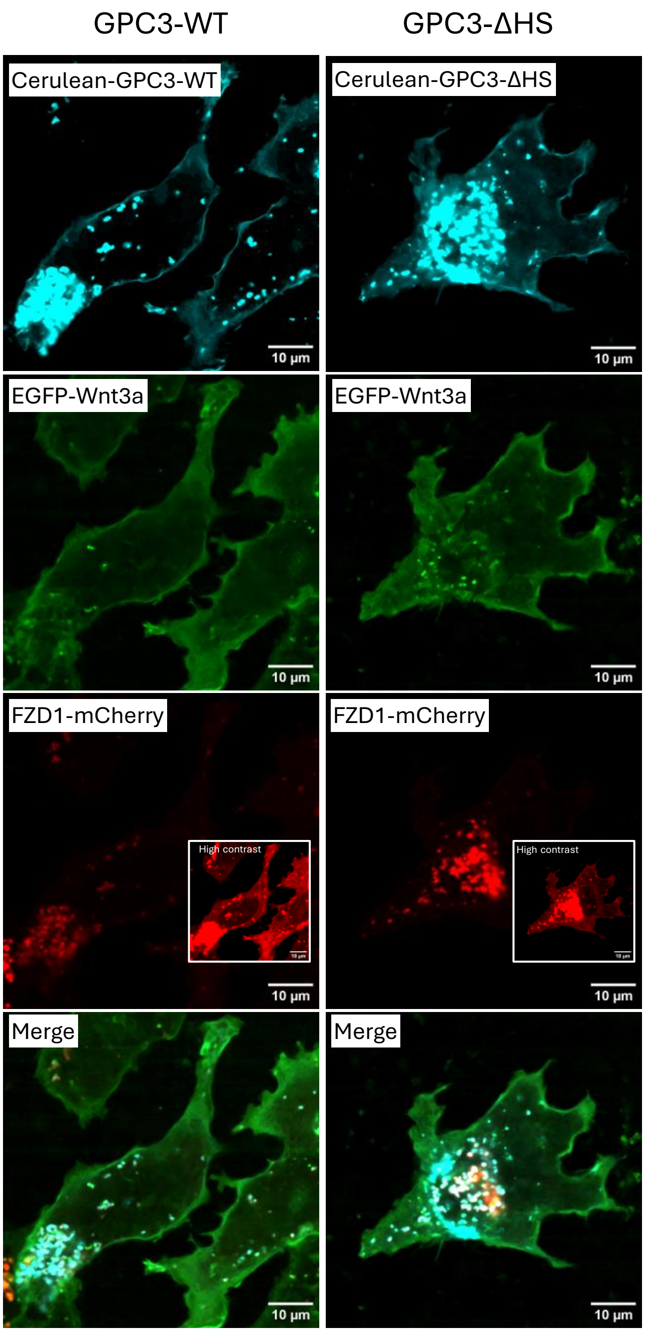
GPC3-WT
GPC3-ΔHS
Cerulean-GPC3-WT
Cerulean-GPC3-ΔHS
EGFP-Wnt3a
EGFP-Wnt3a
FZD1-mCherry
FZD1-mCherry
High contrast
High contrast
Merge
Merge
10 μm

Figure S2

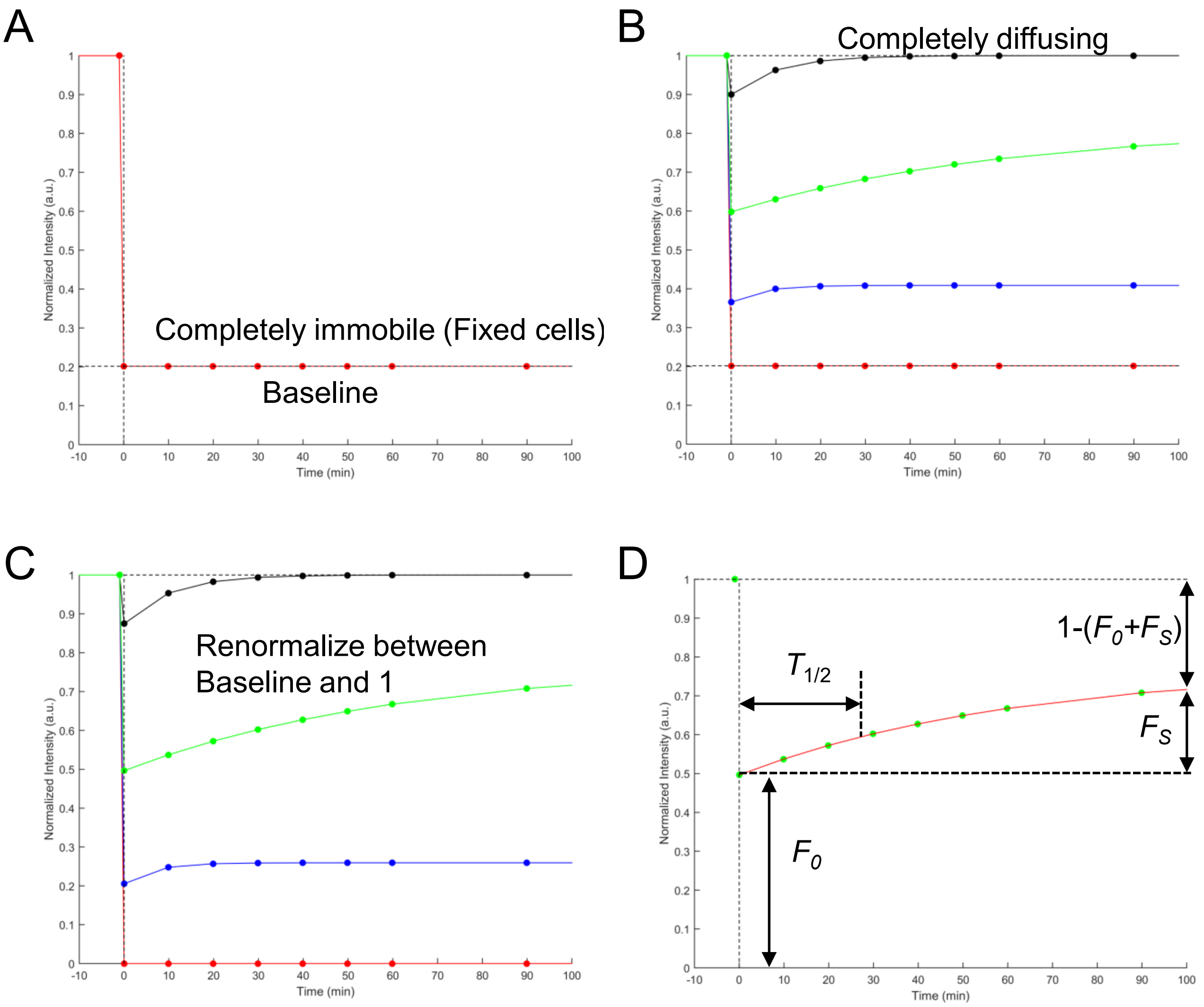
A
Completely immobile (Fixed cells)
Baseline
B
Completely diffusing
C
Renormalize between Baseline and 1
D
1-(F0+FS)
T1/2
FS
F0
Normalized Intensity (a.u.)
Time (min)

Figure S3

A

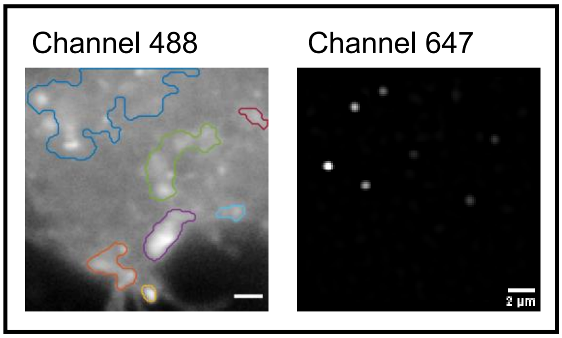

B

	Whole population (%)	High dense molecules (%)	Low dense molecules (%)
Bound Fraction	66.49 ± 0.68	71.39 ± 0.76	66.23 ± 0.73
